# Kleptomania as a neglected disorder in psychiatry

**DOI:** 10.1192/j.eurpsy.2021.176

**Published:** 2021-08-13

**Authors:** J. Torales, A. Ventriglio, I. González, J. Castaldelli-Maia

**Affiliations:** 1 Psychiatry, National University of Asunción, Asunción, Paraguay; 2 Psychiatry, University of Foggia, Foggia, Italy; 3 Psychiatry, University of São Paulo, São Paulo, Brazil

**Keywords:** Shoplifting, Stealing, Impulse control disorders, Kleptomania

## Abstract

Kleptomania is an impulse control disorder characterized by the irresistible urge to steal not for monetary gain. Since its conceptualization, this categorical diagnosis has been conflated with common beliefs regarding the social class and gender such as the idea that women are intrinsically fragile and that people in the middle class were unlikely to commit theft. Also, its use has been controversial in the medical and forensic fields. This presentation will provide a historical excursus through the definitions of the syndrome and summarize the available pharmacological and psychotherapeutic options for its treatment. Currently, there is a lack of systematic studies regarding the clinical characteristics of kleptomania and its treatment options for practical standardized approaches.

**Disclosure:**

No significant relationships.

